# (2-Methyl­quinolin-8-olato-κ^2^ *N*,*O*)di­phenyl­(thio­cyanato-κ*N*)tin(IV)

**DOI:** 10.1107/S1600536809055755

**Published:** 2010-01-16

**Authors:** Ezzatollah Najafi, Mostafa M. Amini, Seik Weng Ng

**Affiliations:** a Department of Chemistry, General Campus, Shahid Beheshti University, Tehran 1983963113, Iran; b Department of Chemistry, University of Malaya, 50603 Kuala Lumpur, Malaysia

## Abstract

The Sn^IV^ atom in the title compound, [Sn(C_6_H_5_)_2_(C_10_H_8_NO)(NCS)], is chelated by the 2-methyl­quinolin-8-olate anion and is five-coordinate in a trigonal-bipramidal geometry [C—Sn—C = 133.47 (13) and 138.77 (12)°]. There are two independent mol­ecules of similar conformation in the asymmetric unit.

## Related literature

The title compound is an unusual example of a diorganotin compound having different anionic groups; for mixed chelated diorganotin compounds, see: Ng (1999 ▶). The diorganotin derivatives of 8-hydroxy­quinolines are bis-­chelate compounds; for the crystal structure of diphenyl­bis(8-hydroxy­quinolinato)tin, see: Linden *et al.* (2005 ▶).
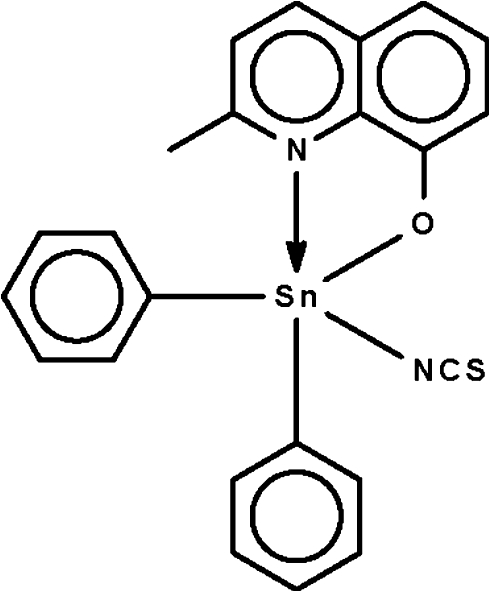


## Experimental

### 

#### Crystal data

[Sn(C_6_H_5_)_2_(C_10_H_8_NO)(NCS)]
*M* *_r_* = 489.14
Triclinic, 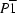

*a* = 9.0093 (5) Å
*b* = 11.5809 (6) Å
*c* = 20.5129 (11) Å
α = 83.7166 (7)°
β = 85.4123 (7)°
γ = 78.2441 (7)°
*V* = 2079.09 (19) Å^3^
*Z* = 4
Mo *K*α radiation
μ = 1.35 mm^−1^
*T* = 295 K
0.40 × 0.20 × 0.20 mm

#### Data collection

Bruker SMART APEX diffractometer
Absorption correction: multi-scan (*SADABS*; Sheldrick, 1996 ▶) *T*_min_ = 0.615, *T*_max_ = 0.775
19942 measured reflections
9461 independent reflections
7796 reflections with *I* > 2σ(*I*)
*R*_int_ = 0.021

#### Refinement

*R*[*F* ^2^ > 2σ(*F* ^2^)] = 0.034
*wR*(*F* ^2^) = 0.103
*S* = 1.04
9461 reflections
507 parameters
H-atom parameters constrained
Δρ_max_ = 0.56 e Å^−3^
Δρ_min_ = −0.96 e Å^−3^

### 

Data collection: *APEX2* (Bruker, 2008 ▶); cell refinement: *SAINT* (Bruker, 2008 ▶); data reduction: *SAINT*; program(s) used to solve structure: *SHELXS97* (Sheldrick, 2008 ▶); program(s) used to refine structure: *SHELXL97* (Sheldrick, 2008 ▶); molecular graphics: *X-SEED* (Barbour, 2001 ▶); software used to prepare material for publication: *publCIF* (Westrip, 2010 ▶).

## Supplementary Material

Crystal structure: contains datablocks global, I. DOI: 10.1107/S1600536809055755/bt5150sup1.cif

Structure factors: contains datablocks I. DOI: 10.1107/S1600536809055755/bt5150Isup2.hkl

Additional supplementary materials:  crystallographic information; 3D view; checkCIF report

## Figures and Tables

**Fig. 1.**
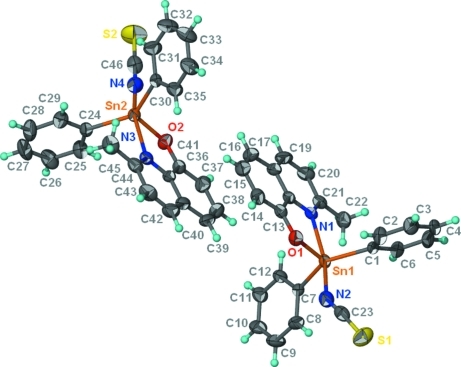
Anisotropic displacement ellipsoid plot (Barbour, 2001) of Sn(NCS)(C6H5)2(C10H8NO); ellipsoids are drawn at the 50% probability level and H atoms of arbitrary radius.

## supplementary crystallographic information

### Experimental

Diphenyltin dichloride (0.34 g,1 mmol), potassium thiocyanate (0.10 g, 1 mol) and 2-methyl-8-hydroxyquinoline (0.16 g, 1 mmol) were dissolved in methanol (10 ml). The white participate was collected and crystallized from dichloromethane; m.p. 466–467 K.

### Refinement

H-atoms were placed in calculated positions (C–H 0.93–0.96 Å) and were included in the refinement in the riding model approximation, with *U*(H) set to 1.2–1.5*U*(C).

### Crystal data

**Table:**

[Sn(C_6_H_5_)_2_(C_10_H_8_NO)(NCS)]	*Z* = 4
*M**_r_* = 489.14	*F*(000) = 976
Triclinic, *P*1	*D*_x_ = 1.563 Mg m^−^^3^
Hall symbol: -P 1	Mo *K*α radiation, λ = 0.71073 Å
*a* = 9.0093 (5) Å	Cell parameters from 9333 reflections
*b* = 11.5809 (6) Å	θ = 2.3–28.3°
*c* = 20.5129 (11) Å	µ = 1.35 mm^−^^1^
α = 83.7166 (7)°	*T* = 295 K
β = 85.4123 (7)°	Prism, yellow
γ = 78.2441 (7)°	0.40 × 0.20 × 0.20 mm
*V* = 2079.09 (19) Å^3^	

### Data collection

**Table:**

Bruker SMART APEX diffractometer	9461 independent reflections
Radiation source: fine-focus sealed tube	7796 reflections with *I* > 2σ(*I*)
graphite	*R*_int_ = 0.021
ω scans	θ_max_ = 27.5°, θ_min_ = 1.0°
Absorption correction: multi-scan (*SADABS*; Sheldrick, 1996)	*h* = −11→11
*T*_min_ = 0.615, *T*_max_ = 0.775	*k* = −15→13
19942 measured reflections	*l* = −26→26

### Refinement

**Table:**

Refinement on *F*^2^	Primary atom site location: structure-invariant direct methods
Least-squares matrix: full	Secondary atom site location: difference Fourier map
*R*[*F*^2^ > 2σ(*F*^2^)] = 0.034	Hydrogen site location: inferred from neighbouring sites
*wR*(*F*^2^) = 0.103	H-atom parameters constrained
*S* = 1.04	*w* = 1/[σ^2^(*F*_o_^2^) + (0.0543*P*)^2^ + 1.4929*P*] where *P* = (*F*_o_^2^ + 2*F*_c_^2^)/3
9461 reflections	(Δ/σ)_max_ = 0.001
507 parameters	Δρ_max_ = 0.56 e Å^−^^3^
0 restraints	Δρ_min_ = −0.96 e Å^−^^3^

### Fractional atomic coordinates and isotropic or equivalent isotropic displacement parameters (Å^2^)

**Table:**

	*x*	*y*	*z*	*U*_iso_*/*U*_eq_	
Sn1	0.82767 (2)	0.23539 (2)	0.420294 (10)	0.03926 (8)	
Sn2	0.09509 (2)	0.75000 (2)	0.091111 (10)	0.03957 (8)	
S1	0.88115 (18)	0.08766 (12)	0.64310 (6)	0.0794 (4)	
S2	0.2261 (2)	0.87393 (15)	−0.13678 (6)	0.0979 (5)	
O1	0.6736 (3)	0.3825 (2)	0.43924 (11)	0.0461 (6)	
O2	0.2507 (3)	0.5987 (2)	0.08617 (11)	0.0485 (6)	
N1	0.8195 (3)	0.3559 (2)	0.32032 (13)	0.0364 (6)	
N2	0.7735 (4)	0.1754 (3)	0.52636 (17)	0.0583 (8)	
N3	0.0928 (3)	0.6567 (3)	0.19826 (13)	0.0409 (6)	
N4	0.1623 (4)	0.7748 (3)	−0.01556 (17)	0.0632 (9)	
C1	1.0470 (3)	0.2573 (3)	0.44053 (15)	0.0362 (6)	
C2	1.0524 (4)	0.3402 (4)	0.48398 (19)	0.0530 (9)	
H2A	0.9628	0.3811	0.5032	0.064*	
C3	1.1910 (5)	0.3624 (4)	0.4989 (2)	0.0619 (11)	
H3A	1.1935	0.4179	0.5281	0.074*	
C4	1.3240 (4)	0.3031 (4)	0.4710 (2)	0.0569 (10)	
H4A	1.4166	0.3189	0.4806	0.068*	
C5	1.3190 (4)	0.2207 (4)	0.4290 (2)	0.0571 (10)	
H5A	1.4092	0.1797	0.4104	0.068*	
C6	1.1814 (4)	0.1968 (3)	0.41342 (18)	0.0473 (8)	
H6A	1.1802	0.1401	0.3848	0.057*	
C7	0.7258 (3)	0.1086 (3)	0.38270 (16)	0.0380 (7)	
C8	0.6944 (5)	0.0112 (4)	0.4234 (2)	0.0568 (10)	
H8A	0.7288	−0.0037	0.4656	0.068*	
C9	0.6119 (6)	−0.0636 (4)	0.4011 (2)	0.0704 (13)	
H9A	0.5898	−0.1277	0.4289	0.085*	
C10	0.5626 (5)	−0.0448 (3)	0.3391 (2)	0.0595 (10)	
H10A	0.5077	−0.0959	0.3246	0.071*	
C11	0.5942 (5)	0.0498 (4)	0.29804 (19)	0.0538 (9)	
H11A	0.5604	0.0630	0.2557	0.065*	
C12	0.6760 (4)	0.1255 (3)	0.31938 (17)	0.0448 (8)	
H12A	0.6981	0.1888	0.2909	0.054*	
C13	0.6292 (4)	0.4647 (3)	0.38955 (16)	0.0398 (7)	
C14	0.5120 (4)	0.5589 (3)	0.39706 (19)	0.0494 (8)	
H14A	0.4597	0.5674	0.4378	0.059*	
C15	0.4700 (5)	0.6433 (3)	0.3433 (2)	0.0578 (10)	
H15A	0.3892	0.7064	0.3491	0.069*	
C16	0.5443 (5)	0.6346 (3)	0.2836 (2)	0.0567 (10)	
H16A	0.5159	0.6920	0.2491	0.068*	
C17	0.6650 (4)	0.5383 (3)	0.27379 (18)	0.0450 (8)	
C18	0.7069 (4)	0.4531 (3)	0.32630 (15)	0.0368 (7)	
C19	0.7480 (5)	0.5186 (4)	0.21314 (19)	0.0561 (10)	
H19A	0.7253	0.5725	0.1766	0.067*	
C20	0.8603 (5)	0.4220 (4)	0.20783 (18)	0.0527 (9)	
H20A	0.9143	0.4102	0.1677	0.063*	
C21	0.8959 (4)	0.3393 (3)	0.26282 (16)	0.0416 (7)	
C22	1.0172 (4)	0.2313 (4)	0.25659 (19)	0.0533 (9)	
H22A	1.0211	0.2072	0.2131	0.080*	
H22B	0.9949	0.1685	0.2881	0.080*	
H22C	1.1134	0.2487	0.2646	0.080*	
C23	0.8225 (4)	0.1396 (4)	0.5705 (2)	0.0530 (9)	
C24	−0.1255 (4)	0.7263 (3)	0.07396 (15)	0.0412 (7)	
C25	−0.1355 (4)	0.6240 (4)	0.04732 (19)	0.0544 (9)	
H25A	−0.0479	0.5682	0.0391	0.065*	
C26	−0.2744 (6)	0.6036 (5)	0.0328 (2)	0.0715 (13)	
H26A	−0.2800	0.5352	0.0140	0.086*	
C27	−0.4036 (5)	0.6845 (5)	0.0460 (2)	0.0766 (14)	
H27A	−0.4973	0.6704	0.0366	0.092*	
C28	−0.3966 (5)	0.7849 (5)	0.0727 (2)	0.0754 (14)	
H28A	−0.4856	0.8388	0.0817	0.091*	
C29	−0.2576 (5)	0.8085 (4)	0.0869 (2)	0.0564 (9)	
H29A	−0.2532	0.8781	0.1048	0.068*	
C30	0.1809 (4)	0.8951 (3)	0.11689 (16)	0.0409 (7)	
C31	0.1905 (5)	0.9914 (4)	0.0717 (2)	0.0618 (11)	
H31A	0.1546	0.9940	0.0302	0.074*	
C32	0.2519 (6)	1.0829 (4)	0.0871 (2)	0.0714 (13)	
H32A	0.2555	1.1477	0.0564	0.086*	
C33	0.3074 (6)	1.0797 (4)	0.1464 (2)	0.0700 (13)	
H33A	0.3533	1.1403	0.1560	0.084*	
C34	0.2960 (6)	0.9872 (4)	0.1924 (2)	0.0678 (12)	
H34A	0.3298	0.9872	0.2341	0.081*	
C35	0.2356 (5)	0.8945 (4)	0.17824 (19)	0.0544 (9)	
H35A	0.2312	0.8309	0.2097	0.065*	
C36	0.2866 (4)	0.5294 (3)	0.14148 (17)	0.0429 (7)	
C37	0.4023 (5)	0.4310 (4)	0.1427 (2)	0.0577 (10)	
H37A	0.4590	0.4112	0.1042	0.069*	
C38	0.4349 (5)	0.3606 (4)	0.2019 (2)	0.0636 (11)	
H38A	0.5139	0.2949	0.2019	0.076*	
C39	0.3555 (5)	0.3851 (4)	0.2585 (2)	0.0641 (12)	
H39A	0.3797	0.3365	0.2969	0.077*	
C40	0.2356 (4)	0.4844 (3)	0.26010 (18)	0.0501 (9)	
C41	0.2027 (4)	0.5574 (3)	0.20114 (16)	0.0395 (7)	
C42	0.1439 (5)	0.5205 (4)	0.3162 (2)	0.0636 (11)	
H42A	0.1599	0.4759	0.3564	0.076*	
C43	0.0345 (5)	0.6176 (4)	0.31239 (18)	0.0625 (11)	
H43A	−0.0252	0.6389	0.3499	0.075*	
C44	0.0077 (4)	0.6889 (4)	0.25189 (17)	0.0490 (9)	
C45	−0.1110 (5)	0.7978 (4)	0.2472 (2)	0.0627 (11)	
H45A	−0.0724	0.8592	0.2200	0.094*	
H45B	−0.1398	0.8228	0.2903	0.094*	
H45C	−0.1981	0.7820	0.2282	0.094*	
C46	0.1877 (5)	0.8159 (4)	−0.0627 (2)	0.0561 (10)	

### Atomic displacement parameters (Å^2^)

**Table:**

	*U*^11^	*U*^22^	*U*^33^	*U*^12^	*U*^13^	*U*^23^
Sn1	0.03234 (12)	0.05092 (15)	0.03605 (12)	−0.01352 (10)	−0.00570 (8)	0.00188 (9)
Sn2	0.03680 (13)	0.04672 (14)	0.03558 (12)	−0.01099 (10)	−0.00574 (9)	0.00222 (9)
S1	0.1024 (10)	0.0763 (8)	0.0585 (7)	−0.0095 (7)	−0.0306 (7)	0.0021 (6)
S2	0.1332 (14)	0.1052 (11)	0.0526 (7)	−0.0328 (10)	0.0259 (8)	−0.0010 (7)
O1	0.0427 (13)	0.0588 (15)	0.0356 (12)	−0.0089 (11)	0.0021 (10)	−0.0039 (10)
O2	0.0483 (14)	0.0534 (15)	0.0393 (12)	−0.0008 (11)	−0.0031 (10)	−0.0020 (11)
N1	0.0357 (13)	0.0382 (14)	0.0378 (14)	−0.0144 (11)	−0.0001 (11)	−0.0024 (11)
N2	0.0534 (19)	0.074 (2)	0.0500 (19)	−0.0213 (17)	−0.0110 (15)	0.0059 (17)
N3	0.0406 (15)	0.0485 (16)	0.0362 (14)	−0.0165 (13)	−0.0022 (11)	−0.0003 (12)
N4	0.060 (2)	0.078 (3)	0.051 (2)	−0.0200 (18)	−0.0103 (16)	0.0147 (18)
C1	0.0325 (15)	0.0390 (17)	0.0372 (16)	−0.0086 (13)	−0.0078 (12)	0.0034 (13)
C2	0.0427 (19)	0.060 (2)	0.057 (2)	−0.0042 (17)	−0.0088 (16)	−0.0172 (18)
C3	0.064 (3)	0.060 (3)	0.069 (3)	−0.017 (2)	−0.026 (2)	−0.012 (2)
C4	0.0391 (19)	0.069 (3)	0.066 (2)	−0.0228 (18)	−0.0220 (17)	0.014 (2)
C5	0.0316 (17)	0.077 (3)	0.055 (2)	−0.0008 (18)	−0.0008 (15)	0.005 (2)
C6	0.0420 (18)	0.052 (2)	0.0474 (19)	−0.0037 (16)	−0.0059 (15)	−0.0088 (16)
C7	0.0309 (15)	0.0376 (17)	0.0440 (17)	−0.0060 (12)	−0.0029 (12)	0.0023 (13)
C8	0.063 (2)	0.055 (2)	0.052 (2)	−0.0188 (19)	−0.0171 (18)	0.0165 (17)
C9	0.090 (3)	0.051 (2)	0.076 (3)	−0.035 (2)	−0.017 (3)	0.017 (2)
C10	0.071 (3)	0.040 (2)	0.073 (3)	−0.0240 (19)	−0.009 (2)	−0.0026 (18)
C11	0.058 (2)	0.059 (2)	0.051 (2)	−0.0237 (19)	−0.0120 (17)	−0.0034 (17)
C12	0.0468 (19)	0.0461 (19)	0.0440 (18)	−0.0190 (15)	−0.0043 (15)	0.0044 (15)
C13	0.0374 (16)	0.0450 (18)	0.0410 (17)	−0.0152 (14)	−0.0038 (13)	−0.0071 (14)
C14	0.0427 (19)	0.051 (2)	0.056 (2)	−0.0100 (16)	0.0009 (16)	−0.0150 (17)
C15	0.051 (2)	0.042 (2)	0.080 (3)	−0.0060 (17)	−0.008 (2)	−0.0092 (19)
C16	0.061 (2)	0.039 (2)	0.069 (3)	−0.0096 (17)	−0.015 (2)	0.0046 (17)
C17	0.0501 (19)	0.0382 (18)	0.0492 (19)	−0.0160 (15)	−0.0083 (15)	0.0032 (14)
C18	0.0361 (16)	0.0390 (17)	0.0393 (16)	−0.0160 (13)	−0.0044 (12)	−0.0033 (13)
C19	0.073 (3)	0.051 (2)	0.046 (2)	−0.023 (2)	−0.0084 (18)	0.0137 (17)
C20	0.065 (2)	0.056 (2)	0.0388 (18)	−0.0210 (19)	0.0064 (16)	0.0014 (16)
C21	0.0429 (18)	0.0479 (19)	0.0370 (16)	−0.0186 (15)	0.0027 (13)	−0.0035 (14)
C22	0.0432 (19)	0.065 (2)	0.049 (2)	−0.0075 (18)	0.0042 (16)	−0.0072 (18)
C23	0.046 (2)	0.056 (2)	0.059 (2)	−0.0172 (17)	0.0110 (18)	−0.0104 (19)
C24	0.0369 (16)	0.053 (2)	0.0337 (15)	−0.0106 (14)	−0.0017 (12)	−0.0007 (14)
C25	0.049 (2)	0.065 (3)	0.051 (2)	−0.0095 (18)	−0.0009 (16)	−0.0199 (18)
C26	0.074 (3)	0.090 (3)	0.065 (3)	−0.040 (3)	−0.008 (2)	−0.022 (2)
C27	0.053 (3)	0.126 (5)	0.061 (3)	−0.043 (3)	−0.010 (2)	−0.004 (3)
C28	0.038 (2)	0.111 (4)	0.071 (3)	−0.005 (2)	−0.0018 (19)	−0.003 (3)
C29	0.051 (2)	0.058 (2)	0.058 (2)	−0.0036 (18)	−0.0050 (18)	−0.0093 (18)
C30	0.0304 (15)	0.0446 (18)	0.0461 (18)	−0.0068 (13)	−0.0026 (13)	0.0013 (14)
C31	0.080 (3)	0.056 (2)	0.052 (2)	−0.022 (2)	−0.016 (2)	0.0096 (18)
C32	0.096 (4)	0.055 (3)	0.068 (3)	−0.034 (2)	−0.003 (2)	0.009 (2)
C33	0.085 (3)	0.061 (3)	0.075 (3)	−0.042 (2)	0.003 (2)	−0.010 (2)
C34	0.080 (3)	0.077 (3)	0.058 (2)	−0.040 (3)	−0.013 (2)	−0.006 (2)
C35	0.061 (2)	0.060 (2)	0.047 (2)	−0.0256 (19)	−0.0108 (17)	0.0061 (17)
C36	0.0414 (17)	0.0419 (18)	0.0479 (19)	−0.0112 (14)	−0.0106 (14)	−0.0045 (15)
C37	0.057 (2)	0.050 (2)	0.067 (3)	−0.0063 (18)	−0.0139 (19)	−0.0097 (19)
C38	0.059 (2)	0.046 (2)	0.084 (3)	−0.0054 (19)	−0.026 (2)	0.003 (2)
C39	0.071 (3)	0.045 (2)	0.078 (3)	−0.022 (2)	−0.034 (2)	0.023 (2)
C40	0.056 (2)	0.049 (2)	0.050 (2)	−0.0235 (17)	−0.0179 (17)	0.0104 (16)
C41	0.0428 (17)	0.0364 (17)	0.0422 (17)	−0.0155 (14)	−0.0128 (14)	0.0056 (13)
C42	0.075 (3)	0.071 (3)	0.048 (2)	−0.031 (2)	−0.012 (2)	0.0167 (19)
C43	0.072 (3)	0.086 (3)	0.0362 (19)	−0.034 (2)	0.0040 (18)	−0.0023 (19)
C44	0.051 (2)	0.062 (2)	0.0389 (18)	−0.0239 (18)	0.0021 (15)	−0.0044 (16)
C45	0.058 (2)	0.077 (3)	0.054 (2)	−0.013 (2)	0.0075 (19)	−0.019 (2)
C46	0.051 (2)	0.067 (3)	0.053 (2)	−0.0124 (19)	−0.0006 (18)	−0.019 (2)

### Geometric parameters (Å, °)

**Table:**

Sn1—O1	2.019 (2)	C17—C18	1.398 (5)
Sn1—C1	2.121 (3)	C17—C19	1.415 (5)
Sn1—C7	2.122 (3)	C19—C20	1.354 (6)
Sn1—N2	2.257 (3)	C19—H19A	0.9300
Sn1—N1	2.348 (3)	C20—C21	1.410 (5)
Sn2—O2	2.015 (2)	C20—H20A	0.9300
Sn2—C30	2.119 (3)	C21—C22	1.494 (5)
Sn2—C24	2.122 (3)	C22—H22A	0.9600
Sn2—N4	2.227 (4)	C22—H22B	0.9600
Sn2—N3	2.341 (3)	C22—H22C	0.9600
S1—C23	1.631 (5)	C24—C25	1.380 (5)
S2—C46	1.634 (5)	C24—C29	1.392 (5)
O1—C13	1.343 (4)	C25—C26	1.380 (6)
O2—C36	1.338 (4)	C25—H25A	0.9300
N1—C21	1.329 (4)	C26—C27	1.367 (7)
N1—C18	1.362 (4)	C26—H26A	0.9300
N2—C23	1.046 (5)	C27—C28	1.352 (7)
N3—C44	1.332 (4)	C27—H27A	0.9300
N3—C41	1.356 (4)	C28—C29	1.394 (6)
N4—C46	1.059 (5)	C28—H28A	0.9300
C1—C6	1.378 (5)	C29—H29A	0.9300
C1—C2	1.390 (5)	C30—C35	1.387 (5)
C2—C3	1.387 (5)	C30—C31	1.383 (5)
C2—H2A	0.9300	C31—C32	1.367 (6)
C3—C4	1.370 (6)	C31—H31A	0.9300
C3—H3A	0.9300	C32—C33	1.346 (7)
C4—C5	1.363 (6)	C32—H32A	0.9300
C4—H4A	0.9300	C33—C34	1.363 (6)
C5—C6	1.392 (5)	C33—H33A	0.9300
C5—H5A	0.9300	C34—C35	1.366 (6)
C6—H6A	0.9300	C34—H34A	0.9300
C7—C12	1.389 (5)	C35—H35A	0.9300
C7—C8	1.392 (5)	C36—C37	1.379 (5)
C8—C9	1.384 (6)	C36—C41	1.419 (5)
C8—H8A	0.9300	C37—C38	1.403 (6)
C9—C10	1.361 (6)	C37—H37A	0.9300
C9—H9A	0.9300	C38—C39	1.340 (7)
C10—C11	1.370 (5)	C38—H38A	0.9300
C10—H10A	0.9300	C39—C40	1.409 (6)
C11—C12	1.379 (5)	C39—H39A	0.9300
C11—H11A	0.9300	C40—C42	1.412 (6)
C12—H12A	0.9300	C40—C41	1.413 (5)
C13—C14	1.367 (5)	C42—C43	1.336 (7)
C13—C18	1.430 (4)	C42—H42A	0.9300
C14—C15	1.413 (6)	C43—C44	1.422 (5)
C14—H14A	0.9300	C43—H43A	0.9300
C15—C16	1.352 (6)	C44—C45	1.478 (6)
C15—H15A	0.9300	C45—H45A	0.9600
C16—C17	1.410 (5)	C45—H45B	0.9600
C16—H16A	0.9300	C45—H45C	0.9600
			
O1—Sn1—C1	109.54 (11)	C20—C19—H19A	119.7
O1—Sn1—C7	111.63 (11)	C17—C19—H19A	119.7
C1—Sn1—C7	138.77 (12)	C19—C20—C21	120.3 (3)
O1—Sn1—N2	83.88 (12)	C19—C20—H20A	119.8
C1—Sn1—N2	92.22 (12)	C21—C20—H20A	119.8
C7—Sn1—N2	94.25 (13)	N1—C21—C20	120.1 (3)
O1—Sn1—N1	75.77 (9)	N1—C21—C22	119.5 (3)
C1—Sn1—N1	94.66 (10)	C20—C21—C22	120.3 (3)
C7—Sn1—N1	93.13 (11)	C21—C22—H22A	109.5
N2—Sn1—N1	159.65 (12)	C21—C22—H22B	109.5
O2—Sn2—C30	114.60 (12)	H22A—C22—H22B	109.5
O2—Sn2—C24	111.83 (12)	C21—C22—H22C	109.5
C30—Sn2—C24	133.47 (13)	H22A—C22—H22C	109.5
O2—Sn2—N4	82.33 (12)	H22B—C22—H22C	109.5
C30—Sn2—N4	95.46 (13)	N2—C23—S1	174.0 (4)
C24—Sn2—N4	93.36 (12)	C25—C24—C29	119.2 (3)
O2—Sn2—N3	75.56 (10)	C25—C24—Sn2	117.1 (3)
C30—Sn2—N3	94.26 (11)	C29—C24—Sn2	123.7 (3)
C24—Sn2—N3	94.28 (11)	C24—C25—C26	120.7 (4)
N4—Sn2—N3	157.88 (13)	C24—C25—H25A	119.7
C13—O1—Sn1	119.2 (2)	C26—C25—H25A	119.7
C36—O2—Sn2	119.2 (2)	C27—C26—C25	119.7 (4)
C21—N1—C18	120.1 (3)	C27—C26—H26A	120.2
C21—N1—Sn1	131.2 (2)	C25—C26—H26A	120.2
C18—N1—Sn1	108.61 (19)	C28—C27—C26	120.6 (4)
C23—N2—Sn1	143.4 (3)	C28—C27—H27A	119.7
C44—N3—C41	120.6 (3)	C26—C27—H27A	119.7
C44—N3—Sn2	130.4 (3)	C27—C28—C29	120.9 (4)
C41—N3—Sn2	108.9 (2)	C27—C28—H28A	119.6
C46—N4—Sn2	161.1 (4)	C29—C28—H28A	119.6
C6—C1—C2	118.8 (3)	C24—C29—C28	118.9 (4)
C6—C1—Sn1	124.8 (2)	C24—C29—H29A	120.5
C2—C1—Sn1	116.4 (2)	C28—C29—H29A	120.5
C3—C2—C1	120.3 (4)	C35—C30—C31	117.8 (3)
C3—C2—H2A	119.8	C35—C30—Sn2	121.4 (3)
C1—C2—H2A	119.8	C31—C30—Sn2	120.7 (3)
C4—C3—C2	120.6 (4)	C32—C31—C30	121.0 (4)
C4—C3—H3A	119.7	C32—C31—H31A	119.5
C2—C3—H3A	119.7	C30—C31—H31A	119.5
C5—C4—C3	119.2 (3)	C33—C32—C31	120.4 (4)
C5—C4—H4A	120.4	C33—C32—H32A	119.8
C3—C4—H4A	120.4	C31—C32—H32A	119.8
C4—C5—C6	121.3 (4)	C32—C33—C34	119.8 (4)
C4—C5—H5A	119.4	C32—C33—H33A	120.1
C6—C5—H5A	119.4	C34—C33—H33A	120.1
C1—C6—C5	119.9 (3)	C35—C34—C33	120.9 (4)
C1—C6—H6A	120.1	C35—C34—H34A	119.5
C5—C6—H6A	120.1	C33—C34—H34A	119.5
C12—C7—C8	118.0 (3)	C34—C35—C30	120.1 (4)
C12—C7—Sn1	121.5 (2)	C34—C35—H35A	120.0
C8—C7—Sn1	120.2 (3)	C30—C35—H35A	120.0
C9—C8—C7	120.1 (4)	O2—C36—C37	122.3 (3)
C9—C8—H8A	120.0	O2—C36—C41	119.2 (3)
C7—C8—H8A	120.0	C37—C36—C41	118.5 (3)
C10—C9—C8	121.0 (4)	C36—C37—C38	120.2 (4)
C10—C9—H9A	119.5	C36—C37—H37A	119.9
C8—C9—H9A	119.5	C38—C37—H37A	119.9
C9—C10—C11	119.7 (4)	C39—C38—C37	122.0 (4)
C9—C10—H10A	120.1	C39—C38—H38A	119.0
C11—C10—H10A	120.1	C37—C38—H38A	119.0
C10—C11—C12	120.2 (4)	C38—C39—C40	120.2 (4)
C10—C11—H11A	119.9	C38—C39—H39A	119.9
C12—C11—H11A	119.9	C40—C39—H39A	119.9
C11—C12—C7	121.0 (3)	C42—C40—C39	126.0 (4)
C11—C12—H12A	119.5	C42—C40—C41	115.4 (4)
C7—C12—H12A	119.5	C39—C40—C41	118.6 (4)
O1—C13—C14	122.5 (3)	N3—C41—C40	122.8 (3)
O1—C13—C18	118.8 (3)	N3—C41—C36	116.7 (3)
C14—C13—C18	118.7 (3)	C40—C41—C36	120.5 (3)
C13—C14—C15	120.3 (4)	C43—C42—C40	121.2 (4)
C13—C14—H14A	119.8	C43—C42—H42A	119.4
C15—C14—H14A	119.8	C40—C42—H42A	119.4
C16—C15—C14	121.5 (4)	C42—C43—C44	121.1 (4)
C16—C15—H15A	119.2	C42—C43—H43A	119.5
C14—C15—H15A	119.2	C44—C43—H43A	119.5
C15—C16—C17	119.8 (4)	N3—C44—C43	118.9 (4)
C15—C16—H16A	120.1	N3—C44—C45	119.4 (3)
C17—C16—H16A	120.1	C43—C44—C45	121.7 (4)
C18—C17—C16	119.4 (3)	C44—C45—H45A	109.5
C18—C17—C19	116.0 (3)	C44—C45—H45B	109.5
C16—C17—C19	124.7 (3)	H45A—C45—H45B	109.5
N1—C18—C17	122.9 (3)	C44—C45—H45C	109.5
N1—C18—C13	116.9 (3)	H45A—C45—H45C	109.5
C17—C18—C13	120.2 (3)	H45B—C45—H45C	109.5
C20—C19—C17	120.7 (3)	N4—C46—S2	177.4 (4)
			
C1—Sn1—O1—C13	97.4 (2)	C19—C17—C18—N1	−0.6 (5)
C7—Sn1—O1—C13	−80.2 (2)	C16—C17—C18—C13	−0.8 (5)
N2—Sn1—O1—C13	−172.4 (2)	C19—C17—C18—C13	−179.7 (3)
N1—Sn1—O1—C13	7.5 (2)	O1—C13—C18—N1	1.9 (4)
C30—Sn2—O2—C36	82.1 (3)	C14—C13—C18—N1	−177.8 (3)
C24—Sn2—O2—C36	−94.8 (3)	O1—C13—C18—C17	−179.0 (3)
N4—Sn2—O2—C36	174.6 (3)	C14—C13—C18—C17	1.3 (5)
N3—Sn2—O2—C36	−6.0 (2)	C18—C17—C19—C20	0.2 (5)
O1—Sn1—N1—C21	178.9 (3)	C16—C17—C19—C20	−178.5 (4)
C1—Sn1—N1—C21	70.0 (3)	C17—C19—C20—C21	0.2 (6)
C7—Sn1—N1—C21	−69.5 (3)	C18—N1—C21—C20	−0.2 (5)
N2—Sn1—N1—C21	179.3 (3)	Sn1—N1—C21—C20	174.4 (2)
O1—Sn1—N1—C18	−5.97 (19)	C18—N1—C21—C22	−178.9 (3)
C1—Sn1—N1—C18	−115.0 (2)	Sn1—N1—C21—C22	−4.3 (5)
C7—Sn1—N1—C18	105.6 (2)	C19—C20—C21—N1	−0.2 (6)
N2—Sn1—N1—C18	−5.6 (4)	C19—C20—C21—C22	178.5 (4)
O1—Sn1—N2—C23	−130.5 (6)	O2—Sn2—C24—C25	−14.3 (3)
C1—Sn1—N2—C23	−21.1 (6)	C30—Sn2—C24—C25	169.6 (3)
C7—Sn1—N2—C23	118.2 (6)	N4—Sn2—C24—C25	68.8 (3)
N1—Sn1—N2—C23	−130.9 (6)	N3—Sn2—C24—C25	−90.4 (3)
O2—Sn2—N3—C44	−177.8 (3)	O2—Sn2—C24—C29	167.0 (3)
C30—Sn2—N3—C44	67.9 (3)	C30—Sn2—C24—C29	−9.2 (4)
C24—Sn2—N3—C44	−66.4 (3)	N4—Sn2—C24—C29	−110.0 (3)
N4—Sn2—N3—C44	−176.2 (3)	N3—Sn2—C24—C29	90.8 (3)
O2—Sn2—N3—C41	4.5 (2)	C29—C24—C25—C26	0.9 (6)
C30—Sn2—N3—C41	−109.9 (2)	Sn2—C24—C25—C26	−178.0 (3)
C24—Sn2—N3—C41	115.9 (2)	C24—C25—C26—C27	−1.3 (7)
N4—Sn2—N3—C41	6.1 (4)	C25—C26—C27—C28	0.6 (8)
O2—Sn2—N4—C46	−144.9 (11)	C26—C27—C28—C29	0.4 (8)
C30—Sn2—N4—C46	−30.8 (11)	C25—C24—C29—C28	0.1 (6)
C24—Sn2—N4—C46	103.5 (11)	Sn2—C24—C29—C28	178.9 (3)
N3—Sn2—N4—C46	−146.5 (10)	C27—C28—C29—C24	−0.8 (7)
O1—Sn1—C1—C6	−156.6 (3)	O2—Sn2—C30—C35	−65.5 (3)
C7—Sn1—C1—C6	20.0 (4)	C24—Sn2—C30—C35	110.6 (3)
N2—Sn1—C1—C6	119.1 (3)	N4—Sn2—C30—C35	−149.5 (3)
N1—Sn1—C1—C6	−80.1 (3)	N3—Sn2—C30—C35	10.6 (3)
O1—Sn1—C1—C2	22.7 (3)	O2—Sn2—C30—C31	111.5 (3)
C7—Sn1—C1—C2	−160.7 (3)	C24—Sn2—C30—C31	−72.4 (4)
N2—Sn1—C1—C2	−61.6 (3)	N4—Sn2—C30—C31	27.5 (3)
N1—Sn1—C1—C2	99.2 (3)	N3—Sn2—C30—C31	−172.4 (3)
C6—C1—C2—C3	0.8 (6)	C35—C30—C31—C32	0.0 (6)
Sn1—C1—C2—C3	−178.5 (3)	Sn2—C30—C31—C32	−177.1 (4)
C1—C2—C3—C4	0.1 (6)	C30—C31—C32—C33	1.4 (8)
C2—C3—C4—C5	−0.9 (7)	C31—C32—C33—C34	−3.0 (8)
C3—C4—C5—C6	0.8 (6)	C32—C33—C34—C35	3.2 (8)
C2—C1—C6—C5	−1.0 (5)	C33—C34—C35—C30	−1.8 (7)
Sn1—C1—C6—C5	178.3 (3)	C31—C30—C35—C34	0.2 (6)
C4—C5—C6—C1	0.2 (6)	Sn2—C30—C35—C34	177.3 (3)
O1—Sn1—C7—C12	72.3 (3)	Sn2—O2—C36—C37	−173.4 (3)
C1—Sn1—C7—C12	−104.4 (3)	Sn2—O2—C36—C41	6.8 (4)
N2—Sn1—C7—C12	157.4 (3)	O2—C36—C37—C38	−179.5 (4)
N1—Sn1—C7—C12	−3.7 (3)	C41—C36—C37—C38	0.2 (6)
O1—Sn1—C7—C8	−101.7 (3)	C36—C37—C38—C39	0.5 (7)
C1—Sn1—C7—C8	81.7 (3)	C37—C38—C39—C40	−0.3 (7)
N2—Sn1—C7—C8	−16.6 (3)	C38—C39—C40—C42	−179.6 (4)
N1—Sn1—C7—C8	−177.6 (3)	C38—C39—C40—C41	−0.7 (6)
C12—C7—C8—C9	−1.9 (6)	C44—N3—C41—C40	−1.3 (5)
Sn1—C7—C8—C9	172.3 (3)	Sn2—N3—C41—C40	176.7 (3)
C7—C8—C9—C10	1.2 (7)	C44—N3—C41—C36	179.4 (3)
C8—C9—C10—C11	−0.3 (8)	Sn2—N3—C41—C36	−2.6 (3)
C9—C10—C11—C12	0.2 (7)	C42—C40—C41—N3	1.2 (5)
C10—C11—C12—C7	−0.9 (6)	C39—C40—C41—N3	−177.8 (3)
C8—C7—C12—C11	1.8 (5)	C42—C40—C41—C36	−179.5 (3)
Sn1—C7—C12—C11	−172.3 (3)	C39—C40—C41—C36	1.5 (5)
Sn1—O1—C13—C14	171.6 (3)	O2—C36—C41—N3	−2.2 (5)
Sn1—O1—C13—C18	−8.1 (4)	C37—C36—C41—N3	178.1 (3)
O1—C13—C14—C15	179.8 (3)	O2—C36—C41—C40	178.5 (3)
C18—C13—C14—C15	−0.5 (5)	C37—C36—C41—C40	−1.2 (5)
C13—C14—C15—C16	−0.7 (6)	C39—C40—C42—C43	178.8 (4)
C14—C15—C16—C17	1.2 (6)	C41—C40—C42—C43	−0.2 (6)
C15—C16—C17—C18	−0.4 (6)	C40—C42—C43—C44	−0.8 (7)
C15—C16—C17—C19	178.3 (4)	C41—N3—C44—C43	0.3 (5)
C21—N1—C18—C17	0.6 (5)	Sn2—N3—C44—C43	−177.2 (3)
Sn1—N1—C18—C17	−175.1 (3)	C41—N3—C44—C45	179.9 (3)
C21—N1—C18—C13	179.7 (3)	Sn2—N3—C44—C45	2.5 (5)
Sn1—N1—C18—C13	4.0 (3)	C42—C43—C44—N3	0.8 (6)
C16—C17—C18—N1	178.2 (3)	C42—C43—C44—C45	−178.9 (4)
